# Complex mediascapes, complex realities: critically engaging with biotechnology debates in Ghana

**DOI:** 10.1080/11287462.2018.1480253

**Published:** 2018-05-31

**Authors:** Joeva Rock

**Affiliations:** Department of Anthropology, American University, Washington, USA

**Keywords:** Biotechnology, genetically modified organisms, Ghana, discourse, ethnography

## Abstract

The recent increase in research and commercialization of genetically modified (GM) crops in Africa has resulted in considerable and understandable interest from farmers, scholars, and practitioners. However, messy situations are often hard to critically engage in from afar, and the recent article published by Braimah et al. [(2017). Debated agronomy: Public discourse and the future of biotechnology policy in Ghana. *Global Bioethics*. doi:10.1080/11287462.2016.1261604] presents certain claims that further obfuscate – rather than clarify – an already complex landscape. In this commentary I first seek to clarify particular details of the Ghanaian “GMO” (as GM crops are colloquially called in Ghana) story with particular focus on certain actors and their stances. Next, I highlight some methodological shortcomings of *Debated Agronomy* and correct certain dubious quotations and claims. Finally, I suggest a more ethnographically and discourse-focused methodology to gain much needed insight into how Ghanaians are actively molding, contesting, and questioning GM discourse, funding, and use.

The recent increase in research and commercialization of genetically modified (GM) crops in Africa has resulted in considerable and understandable interest from farmers, scholars, and practitioners. Within academic circles especially, many of us are students of the “first” Green Revolution, and as such, are eager to understand the complex processes surrounding GM research, promotion and adoption in Africa. I have been studying debates, networks and activism surrounding GM crops in Ghana since 2013, and was thus interested to read *Debated Agronomy: Public Discourse and the Future of Biotechnology Policy in Ghana* (Braimah, Atuoye, Vercillo, Warring, & Luginaah, 2017), one of the sole articles published on Ghana and GM crops thus far (cf. Ignatova, 2015, 2017).

However, the article contains errors, and given that Braimah et al. is one of the few publications on the Ghanaian GM context – and indeed any African GM context – these errors are worth addressing. As I demonstrate in this article, Braimah et al.’s attempt to unravel the “complex and messy nature of the GMO debate in Ghana” (2017, pp. 6–7) through a methodology based solely on news articles further obfuscates, rather than clarify, an already complex landscape. Thus, in this article I use clarifications of Braimah et al. as a launching pad for further interrogating and understanding biotechnology projects across Africa. I first seek to clarify particular details of the Ghanaian “GMO”(as GM crops are colloquially called in Ghana) story with particular focus on certain actors and their stances. Next, I highlight some methodological shortcomings of *Debated Agronomy* and correct certain dubious claims. Finally, I suggest a more ethnographically and discourse-focused methodology to holistically ascertain the complexities of a situation and avoid (re)producing inaccuracies.

Methodological issues are ethical ones, and form the basis of my two-fold critique. First, I argue that relying on media sources to understand a complex situation, as Braimah et al. do, is insufficient. Here, I draw from Arjun Appadurai (1990, p. 9) and use the term *mediascape* to suggest that media houses, such as those sampled in Braimah et al., are not simply entities that report news, but rather, sites where capital, ideology and politics intertwine, and where actors battle for influence. And it is within media formations – television stations, online and print sources, tweets, and so on – where “complex transnational … imaginary landscapes” are constructed and contested (Appadurai, 1990, p. 4). With this understanding, it is imperative to scrutinize media sources as situated and cultural constructs rather than accept them at face value in the discussion of agricultural biotechnology and development.

Second, I argue that the two analytical categories offered by Braimah et al. to understand of Ghanaian GMO debates – locally and internationally produced GMOs – are unstable and misleading. This is evidenced by the research of myself and others investigating similar issues on the African continent; many GM crops currently being researched in African countries are local varieties that have been genetically modified in American or Australian labs (Dowd-Uribe, 2016; Dowd-Uribe & Schnurr, 2016; Ignatova, 2015; Muraguri, 2010; Schnurr & Gore, 2015; Schurman, 2016). Thus, they are neither purely local nor purely global. Accurately analyzing these projects is important for understanding the larger context of the New Green Revolution for Africa, under which proponents seek to radically alter African agricultural production (Davidson, 2012; Gengenbach et al., 2017; Moseley, Schnurr, & Bezner Kerr, 2015).

## Historical clarifications

The crux of Braimah et al.’s analysis rests on the suggestion that there are two types of GMOs in Ghana; *locally produced GMOs*, including those “developed by local companies, institutions and individuals” and “imported from neighbouring countries,” and *international GMOs* that rely on the “involvement of international corporations” (2017, p. 9, 11). These categories form the basis for which Braimah et al. understand GMO debates, finally concluding that, “we found that most of the claims from the pro- GMOs coalition were related to ‘local GMOs’, which probably suggests a strong support for ‘local GMOs’ in the debate” (2017, p. 6). However, currently there are no Ghanaian companies developing GMOs, nor are Ghanaian institutions with the capacity to genetically modify organisms in house. In this section, using ethnographic data and organizational reports, I will briefly outline the history of GM crops in Ghana to demonstrate the international partnerships within which local institutions working on GMOs are enveloped.

The data I present here is from 13 months of ethnographic research in Ghana, including 66 interviews with Ghanaian regulators, scientists, food sovereignty activists and farmers (2014, 2015–2016).^1^ I spent a majority of my time conducting participant observation with activists that make up the Food Sovereignty Platform, an umbrella group of activist and civil society organizations that have been opposing GMOs since 2013. Moreover, following Schnurr and Gore (2015) and Schurman (2016), I sought to understand the complex international donors and actors involved the GMO projects. To that end, I conducted participant observation and semi-structured interviews with Ghanaian scientists and regulators involved in GMO projects and development practitioners. Gate-keepers and snowball sampling allowed me access to internal meetings of the National Biosafety Authority (NBA), NBA-sponsored journalist trainings, workshops for Ghanaian Members of Parliament and extensive interviews with Ghanaians carrying out GMO research, regulation and lobbying. Access to these spaces revealed data not available through media reports and otherwise publically-facing documents, and form the basis of my suggestion that understanding GMO debates in Ghana and elsewhere cannot be accurately – and thus ethically – scrutinized from afar.

Lastly, I relied on organizational reports from agencies involved in GM research and promotion in Ghana, such as annual reports from the United States Department of Agriculture (USDA) Foreign Agricultural Service and the Alliance for a Green Revolution in Africa (AGRA). Such documents revealed, for instance, how US-created intermediaries, such as the US Program for Biosafety Systems (PBS),^2^ sponsor “sensitization” projects for Ghanaian parliamentarians and officials on issues pertaining to biotechnology (USDA, 2011, p. 8).

In Ghana, PBS works alongside the Open Forum for Agricultural Biotechnology (OFAB) to lobby for public and governmental acceptance of GMOs. OFAB was created by the African Agricultural Technology Foundation (AATF), an intermediary organization founded by the Rockefeller Foundation to act as a broker between private industry, African governments, and African state research councils (Schurman, 2016). And it is AATF, not Ghanaian scientists, who brought bt cowpea and nitrogen-use efficient, water-use efficient, salt tolerant (NEWEST) rice to Ghana (Ignatova, 2015).^3^

AATF oversees financial and technical coordination between African scientists and corporate technical partners (Ignatova, 2015). Both the bt cowpea and NEWEST rice projects are comprised of a variety of international actors. For instance, the bt cowpea project is being facilitated in Nigeria and Burkina Faso in addition to Ghana, and Monsanto has provided the bt gene (“AATF Plans”, n.d.). In addition to Ghana, the NEWEST rice project is also operating in Uganda and Nigeria, and Arcadia Biosciences has provided the genes and technical genetic modification; currently no Ghanaian lab possesses the equipment to undertake genetic modification in country (“Nitrogen-Use”, n.d.). At the time of writing, these are the sole GM crops under field trial at various posts within the Ghanaian state Council for Scientific and Industrial Research (CSIR) and there are no GM seeds currently commercially available.

These donors and intermediary organizations are missing from Braimah et al.’s (2017, p. 7) listed pro-GM coalition, likely because their existence and influence are rarely mentioned in Ghanaian media.^4^ However, these details are essential for understanding the history and decision-making behind current GMO projects in Ghana and other African countries. In Kenya, Muraguri found that GMO projects “are science led rather than demand led or user driven … [and] originate outside the national agriculture system and are not linked to national development goals” (2010, p. 300). Similarly, in Uganda, Schnurr and Gore found that the nearly 20 GM crops being researched were all financially supported by international donors (mainly USAID and the Gates and Rockefeller Foundations) and/or agribusiness firms, leading them to similarly conclude as Muraguri (2010) that “demand for experimentation and legislation have not come from farmers … but rather as a result of a large volume of investment” (2015, pp. 60–61, 68).

While the involvement of private donors and private institutions is a bedrock of neoliberal development (Crewe & Harrison, 1998), the instability created by donor networks has dire consequences for GM projects. In Ghana, field trials of bt cotton were suspended after Monsanto, the core gene and financial donor, pulled their funding from a joint project with CSIR (Gakpo, 2017). This decision came after cotton companies in Burkina Faso announced they would no longer grow bt cotton, citing inferior lint quality (Dowd-Uribe & Schnurr, 2016). Without Monsanto funding and support, Ghanaian scientists cannot continue the project; it is Monsanto, not CSIR, whose name lines bureaucratic paperwork, according to a Ghanaian regulator with whom I spoke, and thus, Monsanto would need to release their proprietary technology to CSIR, an unlikely scenario.

As Braimah et al. note, issues related to patents are important for Ghana’s GMO story. However, missing from their analysis is the involvement of major international donors, who have not only provided technical expertise but have also funded efforts to pass legislation and lobby public support of GM crops. One example of these efforts is the *Plant Breeders Bill*, which Braimah et al. describe as “GMO policy” “which would allow [biotechnology] application” and “establish a legal framework to promote the breeding of new plant varieties and improve food production in Ghana” (Braimah et al., 2017, p. 4, 3). However, this characterization is not consistent with the text of the bill. Research into, and regulation of, GM crops was legalized in 2011 with the passing of the Biosafety Act, which established governmental regulation and oversight. This includes the National Biosafety Authority, which has the sole authority to approve or deny applications for GM research and/or commercialization (USDA, 2015).

The Plant Breeders Bill, on the other hand, is an intellectual property rights instrument that would allow plant breeders to apply for “exclusive right[s]” on “new varieties of plants … [marked by] a set of uniform and clearly defined principles” (Plant Breeders Bill, 2013, p. i). As Braimah et al. correctly note (2017, p. 7), proponents of the Plant Breeders Bill publically claim that the Bill is unrelated to GMO field trials and applications (Braimah et al., 2017, p. 7). However, evidence from elsewhere shows how plant breeder patents have grown exponentially and almost in parallel with the growth of biotechnology and agribusiness firms (Boyd, 2003; Cleveland & Murray, 1997). And as Schurman (2016, p. 7, 9) notes, many agribusiness giants were originally reticent to enter African markets, worried that weak or missing intellectual property legislation in many African countries would put their property at risk. Thus, to encourage them, donors – including the Rockefeller Foundation – promised to assist with the policy realm (Schurman, 2016, p. 8).

In Ghana, this assistance first came in the form of liberalizing the seed sector via the Plant and Fertilizer Act (2010). Passed by Parliament a year before the Biosafety Bill, the Plant and Fertilizer Act “allows organizations in the public and private sector to produce foundation seed,” a process formerly restricted to state scientists (National Seed Plan, 2015, p. 10). The Act was spearheaded by international donors, including AGRA’s “policy node[, who] contributed to the development and approval [of the Act] by Cabinet, Parliament and assent by the President” (AGRA, 2017, p. 25).

At a 2016 sensitization workshop for Ghanaian parliamentarians I attended, a Ghanaian scientist told the room that the Plant Breeders Bill had originally been apart of the Plant and Fertilizer Act. However, the Ghanaian attorney general decided that intellectual property rights needed to be dealt with separately, and thus introduced the Plant Breeders Bill in 2013. Without intellectual property rights, the scientist told me on the sidelines of the event, corporate partners would be hesitant to continue their support.

Despite the involvement of “large corporations and organizations” (Braimah et al., 2017, p. 11) in the GMO and policy realms, both Ghanaian and international GMO proponents continue to frame GMOs as “homegrown” solutions (Schurman, 2016). Creating an auspice of GMOs as a “homegrown” solution is a tactic used by GMO proponents in nearly a dozen African countries currently experimenting with GMOs under the New Green Revolution for Africa. This is, as one Ghanaian official described to Ignatova (2015, p. 105), in part a way to obfuscate the involvement of international actors such as Monsanto as to not raise any alarms of foreign interests operating in country, and to create a sort of moral argument that the technology is an African solution to an African problem (Wetzels, 2017) and that GMOs are needed to address African hunger (Stone & Glover, 2016). However, as I have demonstrated here, GMO projects in Ghana (and elsewhere in Africa) rely on a dizzying array of local and international actors.

## Contentious mediascapes

Now that I have clarified the international nature of many of the GMO projects in Africa, I wish to attend to dubious claims that result from methodological issues in Braimah et al. Similar to above, I aim to show how intimate, important details about GMO debates are not discernable from the popular news articles and media pieces sampled in Braimah et al. (2017, pp. 4–5), yet are necessary for understanding the on-the-ground scenario. There are two characteristics of the Ghanaian media landscape that make it hard to “[unravel] the values, ideologies and social structures that inform … claims by the various stakeholders” (Braimah et al., 2017, p. 3).

First, though Ghanaian media houses may be competitive, many media houses publish press releases and pre-written articles by outside authors and organization themselves without editing or fact-checking, and at times, categorizing the text as “news” rather “opinion” or “press release.” This happens frequently with both pro- and anti- GMO organizations, and an article featured in Braimah et al. demonstrates the challenge this poses to ascertaining the veracity of a text. For example, Braimah et al. present text from “an anti-GMO activist and columnist”:

… It is strange that Ghana, Tanzania and Ethiopia have been targeted by the G8 for the accelerated proliferation of GM crops, under the guise of ensuring food security … the appointment of Kofi Annan as AGRA’s chairman was a strategic decision that the Gates Foundation made to silence criticisms that its agricultural development agenda was a White Man’s Dream for Africa. (Braimah et al., 2017, p. 11)

I am familiar with this particular author, a well-known member of Food Sovereignty Ghana, a leading anti-GMO group, and immediately recognized the text *not* as his own. This article was posted to his blog – Pan Africanist International – a day before published in GhanaWeb, and I suspect that he submitted his own post to be published in GhanaWeb rather than being commissioned as a “contributor.” In the original text (both on the blog [Pan-Africanist, 2012] and reproduced in GhanaWeb [Jehu-Appiah, 2012]), “the appointment of Kofi Annan as AGRA’s chairman was a strategic decision that the Gates Foundation made to silence criticisms that its agricultural development agenda was a ‘White Man’s Dream for Africa’” is placed in quotations and righty attributed to a policy report published by the Oakland Institute, a US-based think-tank (Mittal & Moore, 2009, pp. 2–3). However, Braimah et al. have removed these quotation marks, citing the entirety of the text to the Ghanaian activist and author, thus assigning text to the wrong source. Whether deliberate or not, altering the quotation and citation at once conflates and erases GMO critiques.

Second, missing from Braimah et al.’s profile of the Ghanaian media are journalist trainings regularly held by GMO proponents around Ghana. I attended one such training in March 2016, organized by the National Biosafety Authority for a handful of selected Ghanaian journalists. The hosts for the day, the CEO of the National Biosafety Authority, the director of the Biotechnology and Nuclear Agriculture Research Institute,^5^ and a retired scientist, lauded journalists for having an interest in science communications, but also admonished some for printing articles that were not “objective” or “factual.” Nonetheless, organizers emphasized that they “[were] not here to … convince you to be a pro GMO advocate,” but instead to encourage journalists to use “facts” and “objectivity” when reporting. A PBS official told the group, “we don’t have the resources to buy airtime to get information out,” so training journalists to “objectively” and “correctly” report on issues pertaining to biotechnology was essential. As I came to understand from this meeting and others, the professional stakes are high for journalists who attend; some are re-invited to other trainings, high-profile events such as closed-door meetings between scientists and Ghanaian parliamentarians, and at least one Ghanaian journalist was awarded a fellowship to attend communications training at the Cornell Alliance for Science at Cornell University. Such trainings demonstrate how media reports are not neutral, but rather important sites of political and ideological struggle Appadurai (1990).

A second reality of Ghanaian mediascapes, and related to the first, is that articles are often published without being fact checked, leading to the circulation of misinformation. For instance, throughout their article, Braimah et al. cite articles of Ghanaians supposedly discussing genetically modified maize:

… I decided to apply the two [varieties of] maize to see the different … To my surprise, I realized the traditional seeds performed well with good yield, far more than the GM seeds. This made [some of] the farmers in the community decide not to do anything with GM seeds. (Braimah et al., 2017, p. 10)

GM maize – whether bt or otherwise – is not currently grown, tested, or sold in Ghana (and if it in fact is, it is not under the jurisdiction of the National Biosafety Authority), but Braimah et al. never mention this, leading the reader to believe that GM maize is present.^6^ Had Braimah et al. highlighted the lack of GM maize in Ghana, it would have strengthened their argument that actors conflate hybrid and GM seeds, and that Ghanaian farmers have been slow to adopt “improved” varieties (Amanor, 2011; Ragasa et al., 2013).^7^ Similarly, the authors claim that GM food crops, including GM tomatoes, are being exported from neighboring Burkina Faso into Ghana (Braimah et al., 2017, p. 8). However, at the time of the article cited (and up to present), the only GM crop commercially approved and grown in Burkina Faso is bt cotton (Dowd-Uribe & Schnurr, 2016); there is no record of GM tomato there nor in Ghana.

As an anthropologist, I can certainly appreciate and understand how GMOs operate in the imaginaries of peoples around the world, and how conflation between hybrids, GMOs, and other improved seeds occur. Braimah et al. rightly acknowledge this. However, while analyzing mediascapes is important for understanding how critical issues such as GMOs are talked about and circulated, failing to scrutinize the sources or claims themselves risks replicating the same “exaggerated” and dubious claims that actors themselves purport to do. While Braimah et al. point out that Ghanaian actors make unsubstantiated claims, by not interrogating the content of the text they present, they themselves inadvertently reproduce misinformation. Thus, it is ethically essential that researchers scrutinize text used as data rather than simply producing it to avoid further circulating misinformation.

## Methodologies for critical engagement

In their analysis, Braimah et al. conclude that “anti-GMO coalitions refused to resist ‘local GMOs’ but are vigorously fighting … the introduction of ‘international GMOs’” (2017, p. 11). As I have shown, Ghanaian GMO projects –any GM project in Africa facilitated by AATF – cannot be categorized into neat binaries of local or international; they are at once both. However, given the highly polarized controversy over GMOs in Ghana, relying solely on media accounts might give one the impression that such a designation exists. Clarifying details in Braimah et al.’s article broadens our collective understanding of GMOs on the African continent. For instance, given that no Ghanaian laboratory has the ability to genetically modify and thus must rely on corporate partners, it is easier to understand activist concerns. They are not are not acting out of ignorance of local versus global GMOs (Braimah et al., 2017, p. 11), but rather, are making deep political economic critiques of the global development system and Ghana’s development trajectory.

By way of conclusion, I wish to suggest an alternative analytical model to content analysis: a Discourse Historical Approach (DHA) to critical discourse analysis. DHA, as articulated by Ruth Wodak and colleagues, privileges socio-political and historical contexts “within which discursive events are embedded” (Wodak, 2012, p. 529). In particular, DHA is useful for interrogating “front staging” texts – texts designed for public consumption that circulate nationally and internationally – such as the data Braimah et al. presented (Wodak, 2012). To this end, DHA encourages the interrogation of intertexutality and interdiscursivity; in other words, understanding ideologies, discourse, and histories that layer texts. Doing so allows researchers to “[demystify]” and “[decipher]” discourse and ideology and challenge the inconsistencies of the content without “taking a position,” as Braimah et al. wish to avoid (2017, p. 2).

For instance, a DHA approach to Ghanaian news articles pertaining to GMOs would include; (1) scrutinizing the veracity of claims made in articles, (2) researching authors, their viewpoints and professions, (3) placing GMOs in historical, political and social context through the use of additional research (e.g. literature reviews, interviews, etc.), and finally, (4) including this context in the written analysis. In other words, it is not sufficient to present, for instance, an article on GM tomatoes traveling from Burkina Faso to Ghana without noting that GM tomatoes do not exist in either country.

By focusing on this error and others in a single article, I hope to demonstrate the ethical importance of holistic methods for studying issues related to biotechnology and beyond. Without clear context and methodological tools, we risk messy-ing the messy situations we aim to understand (Kingori & Parker, 2017). As researchers with privileged platforms, it is ethically essential to present a holistic picture of the projects and phenomena we wish to study.

## Notes

1 Given the highly contentious political environment concerning GMOs in Ghana, I have anonymized all interviews referenced herein.

2 The Program for Biosafety Systems (PBS) was initiated by the United States (US) Department of State under US President George W. Bush’s Presidential Initiative to End Hunger in Africa (USAID, 2006).

3 Interview with rice plant breeder, CSIR, April 2016.

4 Another core member of the pro-GM coalition is the Ghana National Association of Farmers and Fisherman (GNAFF), who Braimah et al. incorrectly list as an “anti-GMO” group (2017, pp. 8–9; USDA, 2015, p. 9). GNAFF has provided a strategic voice for the pro-GM group, using their status as a farmers association to make claims on behalf of the nation’s farmers (details of their membership base, however, are hazy). They solidified their support for GM crops in 2015 when they joined the Ministry of Food and Agriculture and National Biosafety Authority as co-defendants on a case brought anti-GM activists that sought to halt field trials (Agyeman, 2015). Moreover, the Vice President of GNAFF, John Dziwornu, has trained with the Cornell Alliance for Science at Cornell University and is an ardent and public supporter of GMOs.

5 The institute is housed under the state-run Ghana Atomic Energy Committee, and “does work on crop irradiation and tissue culture but does not have advanced research capacity to conduct genetic modification” (Ignatova, 2015, p. 111).

6 Interview with Ghanaian biotechnology regulator, December 2015.

7 However, the authors did not include a full citation for this article, the radio program cited in the article, nor others cited in their piece, making it difficult to verify the context of articles like these.
